# Analysing the effects of hardware upgrades on the performance of a LABCOM+ clinical laboratory computer system

**DOI:** 10.1155/S1463924687000075

**Published:** 1987

**Authors:** Arthur A. Eggert, Gary J. Smulka, Thomas J. Blankenheim, Kenneth A. Emmerich

**Affiliations:** 1 Department of Pathology and Laboratory Medicine University of Wisconsin Madison Wisconsin 53792 USA; 2 Clinical Laboratories University of Wisconsin Hospital Madison Wisconsin 53792 USA


Journal of Automatic Chemistry/*Journal of Clinical Laboratory Automation*, Vol. 9, No. 1 (January–March 1987), pp. 37–40

### Analysing the effects of hardware upgrades on the performance of a LABCOM+ clinical laboratory computer system

**Arthur A. Eggert,**

*Department of Pathology and Laboratory Medicine, University of Wisconsin, Madison, Wisconsin 53792, USA*

**Gary J. Smulka, Thomas J. Blankenheim and Kenneth A. Emmerich**

*Clinical Laboratories, University of Wisconsin Hospital, Madison, Wisconsin 53792, USA*

The performance of all computer systems is limited by the speed of their components. Moreover, it is rare that anyone believes their computer system's speed is adequate for the tasks to be performed, particularly those using interactive systems. The clinical laboratory is no exception to those areas where speed is important. The difference in the time spent using a system with slow response time and fast response time can be the equivalent of several technologist positions in a large laboratory. Replacing a slower piece of hardware with a faster one is therefore always appealing, but the actual effects of the replacement may be hard to quantitatively evaluate and may not be as substantial as indicated by the manufacturer or as might appear likely from the equipment's specifications. Statements such as 'it seems to run faster' or 'we haven't noticed any change' made by the laboratory staff are not an adequate or necessarily accurate reflection of what has occurred. As a consequence, some type of benchmarking procedure is necessary to give a quantitative measurement of performance changes.

Computer system time can properly be divided into three parts—time spent executing user programs (getting the work done), time spent waiting for peripheral devices to complete their operations, and time spent by the operating system (variously also called 'monitor' or 'queue cycler') to manage the computing environment:

System time = Program time + Queue cycler time + Disk wait time.

The last two may be collectively regarded as overhead [1]. Naturally, the user would like to see 100% of the time spent executing user programs, but this is not possible. The user also desires that the programs be executed as fast as possible; however, the speed is limited by technical and cost considerations. Moreover, there is a fundamental difference between a clinical laboratory environment and a large computer shop environment. In the later case, the system workload will be balanced as much as possible because the operators will load in more batch processing when time-sharing demands are low. In the clinical laboratories this is generally not the case. While some background printing, data retrieval and calculations may be going on, most systems primarily serve the bench workers during the day shift, with major printing and systems functions relegated to off hours. As a result of this, the performance of a clinical laboratory computer system must be measured based on its capacity to provide good response time during the primary shift, with a secondary constraint that it must finish the report printing in the available off-hour time (adequate throughput) [2].

The sum of the queue cycle time, the program time and the disk wait time must equal 100% of the time available during any arbitrary time period. When the system is under maximum load, the queue cycler time is minimized because the cycler will quickly find a user application waiting to run and will transfer control to it. The queue cycler time will therefore represent the time it actually needs to perform its tasks. As the system load lessens, the queue cycler time will increase as it expands to fill the unrequired time. Two measurements of system performance can therefore be formulated.

*Disk-wait/compute-time ratio:* The ratio of the time spent waiting for disk to the time spent executing user programs is called the disk-wait/compute-time ratio. Because the time spent waiting for the disk cannot be used for productive work, it is essentially wasted; therefore, minimizing this ratio is valuable. When this ratio is large, a system is said to be disk bound.

*Queue cycler histogram:* A queue cycler histogram is the frequency of occurrence versus the distribution of queue cycler time percentages. If that diagram shows that the queue cycler time is low a larger percentage of the total

```
1121 06/04/86        %K:  24 %U:  76 PROG:  50 DISK:  19 QCYC:  30 SWAP:   0

Slot Pgm    % Slot Pgm    % Slot Pgm    % Slot Pgm    % Slot Pgm    % Slot Pgm   %

-----------------------------------------------------------------------------------

  1.  RM    0   2.  MI   10   3.  EP    0   4.  EP    1   5.  EP    1   6.  RE   0

  7.        0   8.  IR    9   9.        0  10.        0  11.  EP    0  12.  ME   2

 13.  CE   38  14.        0  15.  ME    1  16.        0  17.        1  18.  AD   0

 19.        0  20.  DE    0  21.  CD    0  22.  TB    0  23.  RE    1  24.       0

 25.  EP  · 0  26.        0  27.        1  28.        0  29.  EP    0  30.       1

 31.        0  32.  RE    3  33.        1  34.  IL    0  35.        1  36.  EP  12

 37.  RE    0  38.        0  39.        0  40.        1  41.  EP    0  42.       0

 43.        1  44.        0  45.  AR    0  46.        1  47.  OC    2  48.  OP   0

 49.  SR    0  50.  QR    2  51.  QA    1  52.  QH    0  53.  XC    1

```

*Figure 1. The Resource Monitor displays the percentage of time spent running each program active as well as the fraction of time spent in the queue cycler, waiting for disk and executing user programs. (Note that %K and %U refer to the percentage modes of the PDP 11/84 operating spaces. PROG, DISK, QCYC and SWAP refer to the percentage of time spent in the applications programs, in waiting for the disk, in the queue cycler and in swapping programs between memory and disk, respectively. Swapping time is routinely zero due to the large amount of memory present.)*

time available, then the system seldom has free time and is overloaded, and more computing power is needed. This is called compute bound. If it is seldom low, then only small improvements will be effected by adding more computing power.

It should be noted that the two measurements are not independent of each other. If the disk-wait/compute-time ratio is high, a heavy occurrence of low queue cycler time might not imply CPU saturation, but, rather, inadequate or improperly used disk resources. In practice, the disk-bound problem must be addressed before the compute-bound problem can be evaluated. By using the methods described below, one can evaluate the status of a laboratory computer system and make appropriate changes.

### Methods and materials

The Clinical Laboratories of the University of Wisconsin Hospital and Clinics run a LABCOM+ system from Laboratory Consulting, Inc. (LCI), of Madison, Wisconsin, USA. LABCOM+ is a self-contained operating system/database management package, but it is general enough that the conclusions drawn should be applicable to other operating system environments on DEC equipment. The software originally ran on a PDP 11/44 with 4 megabytes of main memory (Digital Equipment Corporation [DEC], Maynard, Massachusetts, USA), 3·0 megabytes of solid state disk equivalent (UW Physical Science Laboratory, Stoughton, Wisconsin, USA) and 40 megabytes of moving-head disk storage (four RL-02s). During the course of the project the 11/44 was replaced with a PDP 11/84, and the RL-02s were replaced with an RA-80 (121 megabytes). Most terminals on the system are teleprinters (DEC LA-36s and LA-120s) or unformatted CRTs (ADM 3As, Mime 2As, and Informer 301Ds), although several formatted Hazeltine Exec 10s are also in use. There are two Southern Systems QT 600 line printers, two Hewlett-Packard 7260A card readers, two TI-810 character printers, and a DEC TS11 tape drive. The system has interfaces to the central hospital computer running ACTION 2000 by Shared Medical Systems, to a PDP 11/44 in the Clinical Laboratories running RSX 11M+ by DEC, and to numerous laboratory instruments.

The problem of quantitative analysis of performance improvements became evident to us after the four RL-02 disk system was replaced with an RA-80. Expectations of generally noticeable system performance improvement were not met. After some thought it became apparent that what had been accomplished was to reduce the amount of time the system seemed slow to the users, but that this was not something they were likely to pay much attention to when it didn't occur. A negative had been removed rather than a positive added, which made the evaluation of satisfaction all the more difficult. Since the division of information between the RA-80, the three megabytes of CCD-controlled solid state disk equivalent and the four-megabyte memory had not been optimized, it was decided to prevent future inconclusive changes by developing a method of benchmarking the current status of the system so that the results of future changes could be compared against it.

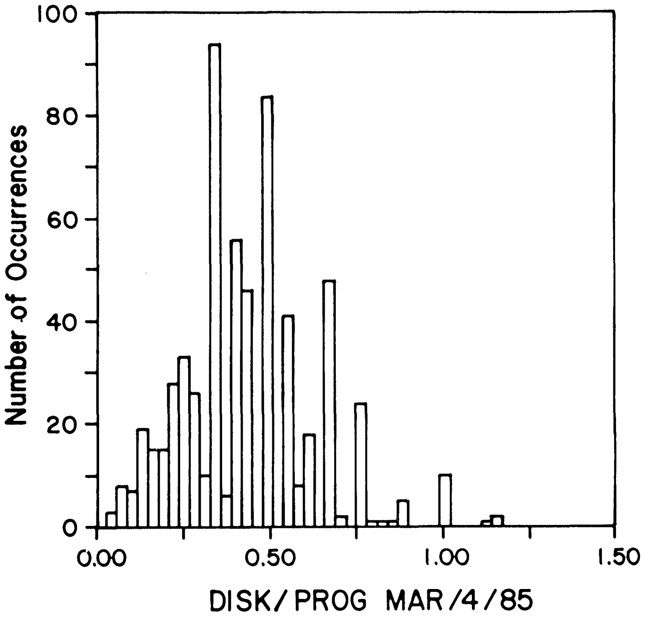

*Figure 2. The PDP 11/44 configuration before optimization had an average disk to compute time ratio of 0·43 running LABCOM+.*

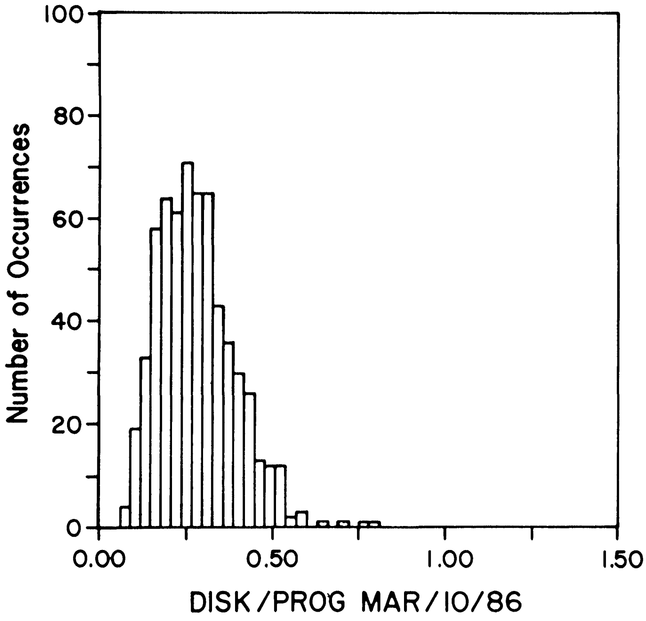

*Figure 3. The PDP 11/44 configuration after optimization showed an average disk to compute time ratio of 0·28 running LABCOM+.*

To evaluate the effects of changes on the system performance, a means of taking numerous snapshots of the system status over the time period of interest was needed. This was accomplished by enhancing a maintenance program called the Resource Monitor (RM), which had previously been developed for LABCOM+ [3]. Among its various operational modes is one which causes the clock interrupt routine to tally in a special memory-resident file what the computer is doing every

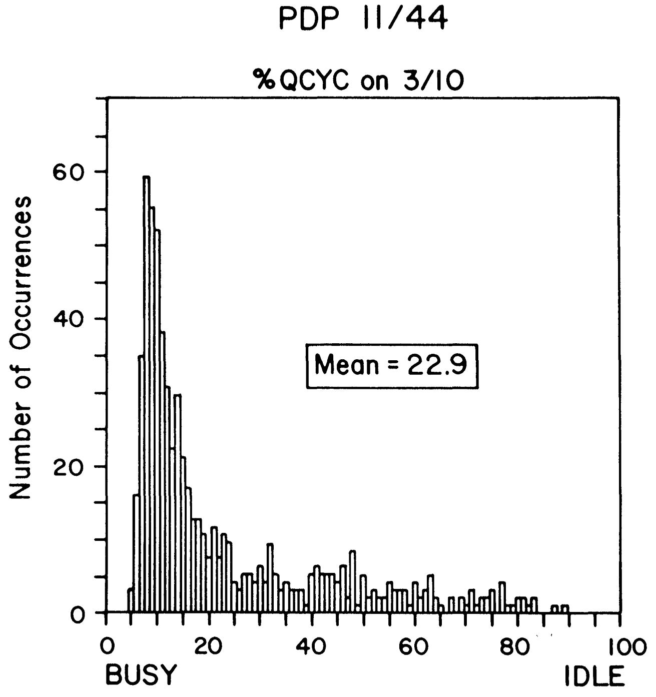

*Figure 4. The PDP 11/44, even with the RA-80, showed a large saturation peak in the queue cycler time usage distribution and a low average idle time. (Note that the mean and the x-axis are in units of per cent.)*

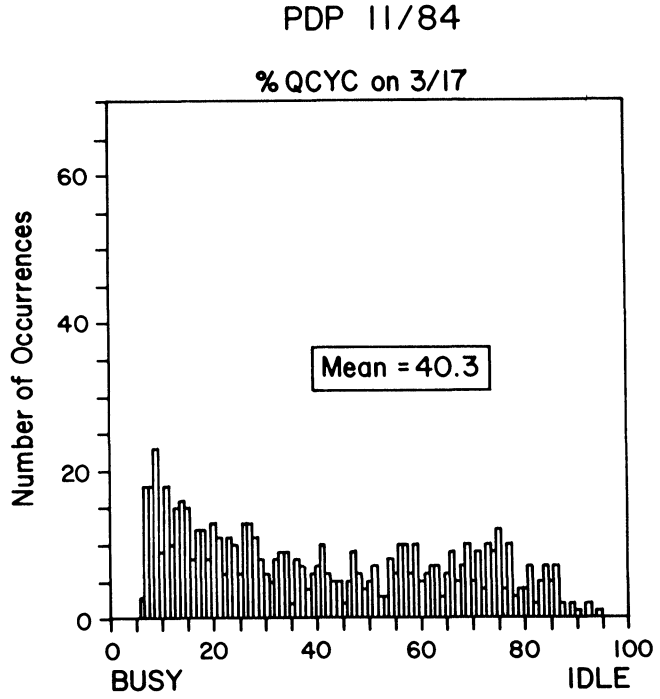

*Figure 5. The PDP 11/84, running with the RA-80, showed a nearly flat distribution of queue cycler time usage and therefore of idle time. (Note that the mean and the x-axis are in units of per cent.)*

one-sixtieth of a second. This file is read and cleared after a specified number of seconds, and the data are displayed on a formatted CRT (figure 1). For these experiments the tally file was read every 60 seconds. While the next set of data was being collected, the formatted CRT (a Hazeltine Exec 10) was printing its display through its printer port to another PDP 11/44 which was running an RSX operating system. The data were decoded by a program running on the latter system and stored in an ASCII file. They were then analysed and graphed using WIGSY (Wisconson Interactive Graphics SYstem), a statistical package originally developed in LINC assembly language in the late 1960s and then converted to Basic for the Apple and redeveloped in Basic 2+ for the RSX system [4]. The quality of the Resource Monitor was improved during the course of the evaluations from a scale in per cent with graduations of 5 and 10% to a scale with graduations in 1%. Figure 3 was rescaled to be easier to compare with figure 2 which has the rougher graduations, while figures 4 and 5 both use the finer graduations.

Two performance evaluations were carried out. The first quantitatively measured what could be accomplished by modifying ('tuning') the operating environment of the system without changing the hardware configuration. The initial benchmark of system performance, measured two weeks after the RA-80 was inserted into the PDP 11/44 system, but before any system optimization was done, was compared with the data gathered a year later from the same configuration after numerous tuning efforts had been performed. While there was no way to guarantee that the loads were identical, the two days used for data gathering were both Mondays in March and were similar in terms of laboratory workload. Review of other data during the last year has shown that the differences in the distributions of percentages on the same date in consecutive weeks is insignificant if holidays or other abnormalities are not present.

The second evaluation compared the changing of hardware while making no other adjustments in the operating environment of the system. The same procedure of data gathering was used, this time on two consecutive Mondays, with the 11/44 being replaced with an 11/84 in the interim. Once again, the workloads appeared to be effectively equal. The disk system used for the second evaluation was the RA-80.

### Results and discussion

In retrospect, it would have been desirable to have benchmarked the system before the introduction of the RA-80, so the actual performance changes could have been documented. On the other hand, it was the failure to have made such a benchmark that led to the two studies reported here. The user response to the disk upgrade clearly indicated that the performance improvement goals had not been met. This led to the development of the benchmarking procedure described above and encouraged the optimization effort. The two major portions of the tuning involved queuing procedure and data allocation between devices. LABCOM+ switches between programs based on the number of disk transfers initiated by a program, on having to wait for a non-disk device to become available or ready, and on waiting for a buffer to fill or empty. Since different disks run at different speeds and all of them run slower then the solid state disk equivalent which, in turn, is slower than the main memory, it is necessary to adjust the number of information transfers that can be made with each device before the system switches to another program. Experimentation was needed to weigh the time expense of information transfers between the computer and the various peripherals to find the best performance. On the other hand, where the data were located was also important. By rearranging the location of the files and reallocating them among the main memory, the solid-state disk and the RA-80, it was possible to place files that were more frequently used onto faster devices while also reducing head sluing time on the disk.

As can be seen from figures 2 and 3, these changes to the way the system managed the disk transfers and to the data organization significantly changed the disk-wait/compute-time ratio from an average of 0·43 to an average of 0·28. This 35% reduction in disk-wait/compute-time ratio represents a year of progressive refinements and system tuning. One may also look at the percentage of user program time both before and after an optimization step or the installation of new equipment. During the measured periods the user programs averaged 44% of the available machine time before optimization and 60% after. The ratio of the difference of the two numbers to the first gives the performance improvement in terms of time available for user programs, in this case, 36%. This is virtually the same as the disk-wait/compute-time ratio. This is somewhat surprising considering that the drop in the percentage of disk time was only 6·5%, from 17·9% to 16·7% of the total time. The most likely explanation of this is that when the disk was frequently disk-bound at peak times, the users resorted to alternate manners of interaction with the computer, which only further compounded the problem. The net effect was that a relatively small improvement in the disk-bound situation caused a dramatic improvement in system performance. The same type of improvement response might be expected from simply replacing a slower disk with a faster one. Before purchase of a new disk system, therefore, it would be well to evaluate it by borrowing a unit like the one being considered and performing the analysis described above to determine if the performance increase will justify the purchase. As can be seen from figures 2 and 3, however, sometimes a significant improvement can be obtained simply by reallocating the storage of data properly.

The results of the second evaluation are illustrated by figures 4 and 5, which show a striking change in distribution of the queue cycle percentage with the introduction of the PDP 11/84. To interpret the results properly, one must realize that our installation of the LABCOM+ system requires 6 to 7% queue cycler overhead even when operating at full capacity. Therefore, the percentage distribution peak for the 11/44 is indeed as far to the left as it can get. Moreover, to find how much unused time (mean idle time) the system has, the measured queue cycler time must be reduced by eliminating the required queue cycler time if accurate comparisons are to be made. The real mean idle time on the 11/44 then is between 16 and 17%. This magnitude of idle time would be adequate for a batch processor where the real time of users is not spent waiting for the system to respond, but it is inadequate for a time-sharing system because the uneven distribution of work causes backlogs when numerous users seek access simultaneously. When the processor is saturated or nearly saturated, as it is a significant percentage of the time in figure 4, response time will lengthen perceptibly for users, a fact that correlates with the qualitative feelings of the technologists. On the 11/84, the actual average idle time was 34% or twice as great as on the 11/44. In addition, the saturation peak was dramatically reduced.

The approach we used could be adapted for the evaluation of new hardware or new data storage schemes for any clinical laboratory computer system. Every system has, or could have, a method of recording what it was doing at numerous, equally spaced intervals each second. If this were transmitted to another computer, it could be stored, analysed and graphed. The receiving computer could be any microcomputer running standard packaged software instead of a larger system as we used. By asking vendors to bring in their products and test them under similar loads of actual operation in the laboratory, the cost versus benefit of upgrades could easily be evaluated.

### References

1. Shaw, A. C., *The Logical Design of Operating Systems* (Prentice-Hall, Inc., Englewood Cliffs, New Jersey, 1974), pp. 198–202.
2. Dowdy, L. W., Eager, D. L., Gordon, K. D. and Saxton, L. V., *Inf. Process. Lett.*, **19** (1984), 209–212.
3. *LABCOM+ User Manual* (Laboratory Consulting, Inc., Madison, Wisconsin, 1986).
4. Cembrowski, G. S., Chandler, E. and Miller, R., *Wisconsin Graphics System* (University of Wisconsin Hospital, Madison, Wisconsin, 1982).

